# A *Plasmodium falciparum C*-mannosyltransferase is dispensable for parasite asexual blood stage development

**DOI:** 10.1017/S0031182019001380

**Published:** 2019-10-23

**Authors:** Borja López-Gutiérrez, Marta Cova, Luis Izquierdo

**Affiliations:** ISGlobal, Barcelona Ctr. Int. Health Res. (CRESIB), Hospital Clínic – Universitat de Barcelona, Barcelona, Spain

**Keywords:** *C*-mannosylation, glycosylation, malaria, *Plasmodium falciparum*, thrombospondin type 1 repeat (TSR) domains

## Abstract

*C-*mannosylation was recently identified in the thrombospondin-related anonymous protein (TRAP) from *Plasmodium falciparum* salivary gland sporozoites. A candidate *P. falciparum C*-mannosyltransferase (*Pf*DPY-19) was demonstrated to modify thrombospondin type 1 repeat (TSR) domains *in vitro*, exhibiting a different acceptor specificity than their mammalian counterparts. According to the described minimal acceptor of *Pf*DPY19, several TSR domain-containing proteins of *P. falciparum* could be *C*-mannosylated *in vivo*. However, the relevance of this protein modification for the parasite viability remains unknown. In the present study, we used CRISPR/Cas9 technology to generate a *Pf*DPY19 null mutant, demonstrating that this glycosyltransferase is not essential for the asexual blood development of the parasite. *Pf*DPY19 gene disruption was not associated with a growth phenotype, not even under endoplasmic reticulum-stressing conditions that could impair protein folding. The data presented in this work strongly suggest that *Pf*DPY19 is unlikely to play a critical role in the asexual blood stages of the parasite, at least under *in vitro* conditions.

## Introduction

Malaria remains one of the most serious global infectious diseases. Only in 2017, it was responsible for 219 million cases and 435 000 deaths worldwide (World Health Organization, 2018). It is generally accepted that malaria elimination from the remaining endemic countries will not be possible with currently available strategies (Alonso *et al*., 2011). In this regard, a better understanding of the numerous gaps of knowledge in the complex biology of the parasite would facilitate the rationale design of new interventions.

The thrombospondin type 1 repeat (TSR) superfamily is a diverse group of proteins often involved in interactions with the extracellular matrix (Tucker, 2004; Morahan *et al*., 2009). Particularly, the functional characterization of *P. falciparum* TSR domain-containing proteins revealed the essential roles that these effectors play in processes involved in cell invasion, egression and transmission of the parasite through the mosquito stages. Thus, circumsporozoite- and TRAP-related protein (CTRP) is essential for ookinete invasion of the mosquito midgut epithelium and oocyst development (Dessens *et al*., 1999), whereas thrombospondin-related protein 1 is required for the subsequent sporozoite egress from oocysts (Klug and Frischknecht, 2017). TRAP, circumsporozoite protein (CSP) and TRAP-related protein are all involved in the sporozoite invasion of the mosquito salivary glands; in the vertebrate host, CSP and TRAP also bind to the hepatocyte surface during sporozoite invasion (Ménard *et al*., 1997; Sultan *et al*., 1997; Combe *et al*., 2009). Finally, TRAP-like protein and thrombospondin-related sporozoite protein have been described to play an important role in hepatocyte cell traversal (Labaied *et al*., 2007; Moreira *et al*., 2008). Other TSR domain-containing proteins are expressed in the blood stages of the parasite. Previously described to be essential for the merozoite invasion of erythrocytes (Baum *et al*., 2006), more recent studies revealed that the merozoite TRAP family protein (MTRAP) is completely dispensable for the viability of asexual parasites. Instead, MTRAP was demonstrated to be essential for gametocyte egress and, thus, for transmission to the mosquito (Bargieri *et al*., 2016). On the other hand, the thrombospondin-related apical merozoite protein (TRAMP) seems to be involved in the invasion of erythrocytes, since antibodies raised against this protein block merozoite invasion (Siddiqui *et al*., 2013). Interestingly, a secreted protein with altered thrombospondin repeat domain (SPATR) is expressed at multiple stages of the parasite such as sporozoites, asexual blood stages and gametocytes. Antibodies raised against SPATR block hepatocyte invasion by sporozoites; however, the biological relevance of SPATR during other stages of the parasite has not been addressed (Chattopadhyay *et al*., 2003).

The elucidation of the crystal structure of TSR domains revealed an unusual three-stranded fold known as the tryptophan ladder, also found in type 1 cytokine receptors and characterized by a motif of stacked aromatic and basic amino acids stabilized by disulfide bonds (Tan *et al*., 2002; Olsen and Kragelund, 2014). In other organisms, TSR domains can be modified by two types of glycosylation, namely *O-*fucosylation and *C*-mannosylation. The protein *O-*fucosyltransferase 2 (PoFUT2) catalyses the *O*-fucose modification of TSR domains, which in turn may be elongated by a *β*3-glucosyltransferase (Kozma *et al*., 2006; Luo *et al*., 2006). On the other hand, DPY19 enzymes have been identified as the glycosyltransferases responsible for TSR *C*-mannosylation (Buettner *et al*., 2013; Shcherbakova *et al*., 2017). Recent works revealed that TRAP and CSP are both modified *in vivo* by a deoxyhexose and a hexose (presumably forming a glucose-*β*1,3-fucose *O*-glycan) in *P. falciparum* and *P. vivax* salivary gland sporozoites. Additionally, TRAP was also shown to be modified by an additional hexose (presumably *C*-linked mannose) in *P. falciparum* (Swearingen *et al*., 2016) but, interestingly, not in *P. vivax* (Swearingen *et al*., 2017). These, and other works (Sanz *et al*., 2013; Cova *et al*., 2015; Bandini *et al*., 2019), are renewing the interest of the *Plasmodium* research community on the extent and relevance of the parasite glycosylation.

*Pf*PoFUT2 was demonstrated to be dispensable for the asexual and sexual blood stage development of the parasite, in agreement with the absence of predicted acceptors with the TSR *O-*fucosylation consensus sequence (Lopaticki *et al*., 2017; Sanz *et al*., 2019). On the contrary, the relevance of *Pf*PoFUT2 for the establishment of the infection in the mosquito host remains a controversial issue. While Lopaticki *et al*. reported that *Pf*PoFUT2 gene disruption resulted in a deficient ookinete invasion and a reduced sporozoite gliding motility and cell traversal activity, Sanz *et al*. did not observe any evident phenotype secondary to PoFUT2 ablation, neither in *P. falciparum* nor in the murine model *P. berghei*. Interestingly, the phenotype described by Lopaticki *et al*. was attributed to a deficient secretion of certain PoFUT2 acceptors, in accordance with the proposed role of *O-*fucosylation in a protein folding quality control system (Vasudevan and Haltiwanger, 2014). However, to our knowledge, the relevance of the *C*-mannosylation of *P. falciparum* TSR-containing proteins has not been addressed before.

*Pf*DPY19 was recently demonstrated to harbour a *C-*mannosyltransferase activity *in vitro* (Hoppe *et al*., 2018). Interestingly, *Pf*DPY19 exhibited a different acceptor specificity than the mammalian enzymes (see Table 1 for a list of putative *Pf*DPY19 acceptors). In other organisms, *C*-mannosylation has been shown to be required for the efficient secretion of certain acceptors, in accordance with the role played by this modification in the stabilization of the tryptophan ladder (Buettner *et al*., 2013; Shcherbakova *et al*., 2017). Taking into consideration the proposed roles for some of the putative acceptors of *Pf*DPY19 in the asexual blood stages, we aimed to describe the relevance of the gene for the viability of *P. falciparum* by disrupting it and analysing the growth phenotype of Δ*Pf*DPY19 mutant parasites.

**Table 1.** Conservation of the apicomplexan DPY19 *C*-mannosyltransferase consensus sequence among TSR domain-containing proteins expressed in *P. falciparum*

	Protein	Gene ID	W	X	X	W	X	X	C	*
Sporozoite	TRAP	PF3D7_1335900	**W**	D	E	**W**	S	P	**C**	Y
	TLP	PF3D7_0616500	**W**	S	E	**W**	S	P	**C**	Y
	TRSP	PF3D7_0104000	**W**	S	E	**W**	S	A	**C**	Y
	TRP1	PF3D7_0822700	**W**	S	E	**W**	S	T	**C**	Y
	CSP	PF3D7_0304600	S	T	E	**W**	S	P	**C**	N
	TREP	PF3D7_1442600	**W**	S	E	**W**	S	G	P	N
Ookinete	CTRP	PF3D7_0315200	**W**	E	E	**W**	S	P	**C**	Y
			F	G	E	**W**	S	E	**C**	N
			**W**	S	D	**W**	S	S	**C**	Y
			**W**	E	E	**W**	N	E	W	N
			**W**	S	D	**W**	S	E	**C**	Y
			**W**	E	T	**W**	V	E	**C**	Y
			**W**	E	E	**W**	G	D	**C**	Y
Blood	MTRAP	PF3D7_1028700	**W**	S	E	**W**	S	A	**C**	Y
			**W**	G	E	**W**	S	E	**C**	Y
	SPATR	PF3D7_0212600	**W**	S	D	**W**	S	A	**C**	Y
	PTRAMP	PF3D7_1218000	**W**	G	E	**W**	S	N	**C**	Y

CSP, circumsporozoite protein; CTRP, circumsporozoite and TRAP-related protein; MTRAP, merozoite TRAP-like protein; PTRAMP, *Plasmodium* thrombospondin-related apical merozoite protein; SPATR, secreted protein with altered thrombospondin repeat; TLP, TRAP-like protein; TRAP, thrombospondin-related anonymous protein; TREP, TRAP-related protein; TRP1, thrombospondin-related protein 1; TRSP, thrombospondin-related sporozoite protein. Residues of the apicomplexan *C*-mannosylation consensus sequence are marked in bold, when conserved.

*TSR domains with or without a conserved *C*-mannosylation consensus sequence are marked as Y and N, respectively.

## Material and methods

### Construction of plasmids for CRISPR/Cas9-mediated PfDPY19 knockout

A single guide RNA (sgRNA) targeting the *Pf*DPY19 genomic locus (PF3D7_0806200) was designed with the Eukaryotic Pathogen gRNA Design Tool (Peng and Tarleton, 2015). To generate the plasmid expressing the *Streptococcus pyogenes* Cas9 and the sgRNA, the primers 5′-TAAGTATATAATATTTAAATTTAGGCCTTTCCATAGTTTTAGAGCTAGAA-3′ and 5′-TTCTAGCTCTAAAACTATGGAAAGGCCTAAATTTAAATATTATATACTTA-3′ were annealed and ligated into a BbsI-digested pDC2-Cas9-hDHFRyFCU plasmid (a generous gift from Ellen Knuepfer) (Knuepfer *et al*., 2017). To generate the rescue plasmid, two homology regions for *Pf*DPY19 of ~530 bp were amplified from *Pf*3D7 1.2B genomic DNA with the primer pairs 5′-ATTCGAGCTCGGTACCCGGGAAATTATGTTGAGCAGAAACTTTCC-3′/5′CAGGTCGACTCTAGAGGATCCACGTCTCGAGCCCCAAGAATAATTTCTAAAAACAT-3′ and 5′-TTCTTGGGGCTCGAGACGTGCGGCCGCATTATATGTTTTCAGCTTGTCTTCC-3′/5′-CAGGTCGACTCTAGAGGATCCGCAATATGGAATATTACTTCCACAT-3′ and were cloned into a pUC19 backbone.

### Parasite culture and transfection

**Fig. 1.**
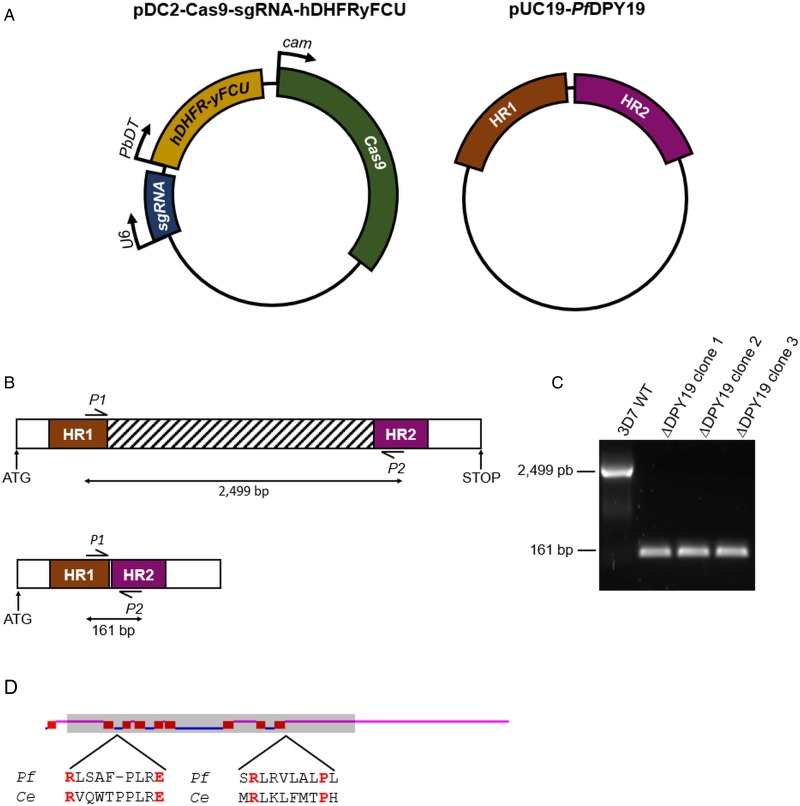
*Pf*DPY19 gene disruption. (A) The pDC2-Cas9-sgRNA-hDHFRyFCU expresses the *S. pyogenes* Cas9 endonuclease, the sgRNA targeting the *Pf*DPY19 genomic locus and a fusion protein of the positive selectable marker human dihydrofolate reductase (hDHFR) and the negative selectable marker yeast cytosine deaminase/uridyl phosphoribosyl transferase (yFCU). The rescue plasmid (pUC19-*Pf*DPY19) contains two homology regions of approximately 530 bp corresponding to the 5′ and 3′ ends of the *Pf*DPY19 gene (PF3D7_0806200). (B) Schematic representation of *Pf*DPY19 wild-type (up) and modified loci (down). In total, 2365 bp of the *Pf*DPY19 coding region (which contain the locus targeted by the designed sgRNA) are excised after double homologous recombination with homology region 1 (HR1) and 2 (HR2) from the rescue plasmid. (C) Polymerase chain reaction (PCR) screening of *Pf*DPY19 disruption of wild-type parasites (3D7 WT) and three different Δ*Pf*DPY19 clones obtained by limited dilution. A negative control reaction (NC) was performed in the absence of template DNA to discard unspecific amplifications. (D) Predicted transmembrane helices (red boxes) of *Pf*DPY19 protein with the TMHMM Server v. 2.0 software (Krogh *et al*., 2001). Loops predicted to localize in the lumen of the ER and the cytoplasm are marked in pink and blue, respectively. The shaded area indicates the deleted gene fragment in Δ*Pf*DPY19 mutants. Amino acids predicted to bind to dolichol-phosphate mannose in *C. elegans* (*Ce*) DPY19 are marked in red and are conserved in the *P. falciparum* (*Pf*) homologue (Buettner *et al*., 2013).

*Plasmodium falciparum* 3D7 1.2B line (kindly provided by Cortés) (Cortés, 2005) was used for transfection and parasite maintenance. *Pf*3D7 1.2B was cultured with B+ human erythrocytes in Roswell Park Memorial Institute (RPMI) medium supplemented with Albumax-II at 37 °C under an atmosphere of 92% N_2_, 3% O_2_ and 5% CO_2_, following standard methods (Trager and Jensen, 1976). For *Pf*DPY19 gene disruption, 60 *µ*g of pDC2-Cas9-sgRNA-hDHFRyFCU plasmids and 15 *µ*g of ScaI-linearized pUC19-*Pf*DPY19 plasmids (Fig. 1A) were transfected into Percoll-purified segmented schizonts by electroporation with the Amaxa 4D system, as previously described (Moon *et al*., 2013). Drug selection (*i.e.* 10 nm WR99210 from Jacobus Pharmaceuticals) was first applied ~20 h post-transfection and maintained for 4 days with daily media changes. The emergence of resistant parasites was monitored by visualizing Giemsa-stained blood smears by light microscopy. Viable parasites were screened by PCR for *Pf*DPY19 gene disruption and treated for 1 week with 1 *µ*m 5-fluorocytosine for negative selection of parasites containing the pDC2-Cas9-sgRNA-hDHFRyFCU plasmid, prior to subcloning by limiting dilution.

### *In vitro* growth assay of parasite asexual development

To assess the biological relevance of *Pf*DPY19 for the asexual blood stage development of the parasite, the growth of two independent clones of *Pf*DPY19 null mutants was compared with that of the wild-type parental line in three biological replicates. Cultures were synchronized at ring stages by sorbitol synchronization and parasitaemias were determined by flow cytometry (FACSCalibur, BD Biosciences, Franklin Lakes, NJ, USA) using the Syto 11 dye to discriminate between infected and uninfected erythrocytes, as described elsewhere (Urbán *et al*., 2011). Cultures were adjusted to ~1.5% parasitaemia and 1% haematocrit. After incubation for one complete intraerythrocytic cycle (~48 h), parasitaemias were determined again by flow cytometry. Multiplication rates for each strain were calculated as the ratio between the final and the initial parasitaemia.

### Oxidative stress induction and parasite survival assay

To assess the ability of *Pf*DPY19 null mutants to cope with the endoplasmic reticulum (ER)-stressing conditions, parasite cultures were subjected to different concentrations of dithiothreitol (DTT) as an oxidative stress proxy. Cultures were treated with DTT at a final concentration of 0.05 and 0.15 M or, alternatively, with phosphate buffered saline (PBS) as a negative control. After ~48 h of incubation, parasitaemia was determined by flow cytometry as described previously. The survival rate for each strain was expressed as the ratio between the parasitaemia of the cultures treated with DTT and the negative controls incubated with PBS.

## Results

### PfDPY19 is not essential for the asexual blood development of P. falciparum

To investigate the relevance of the *Pf*DPY19 *C*-mannosyltransferase, we disrupted the gene coding for this glycosyltransferase (Fig. 1). As demonstrated by PCR, *Pf*DPY19 gene was truncated in edited parasites. Remarkably, the deleted gene fragment (see striped box in Fig. 1B) codes for eight out of the nine predicted transmembrane domains of the native protein. Moreover, the delated fragment also codes for the conserved amino acids that have been predicted to bind to the dolichol-phosphate mannose precursor (Buettner *et al*., 2013). Given the magnitude of the gene deletion, it is highly unlikely that the generated *Pf*DPY19 null mutants could yield a functional *C*-mannosyltransferase activity. Thus, the viability of these mutants strongly suggests that *Pf*DPY19 is not essential for the asexual blood stage development of the parasite.

### PfDPY19 gene disruption does not affect parasite growth

**Fig. 2.**
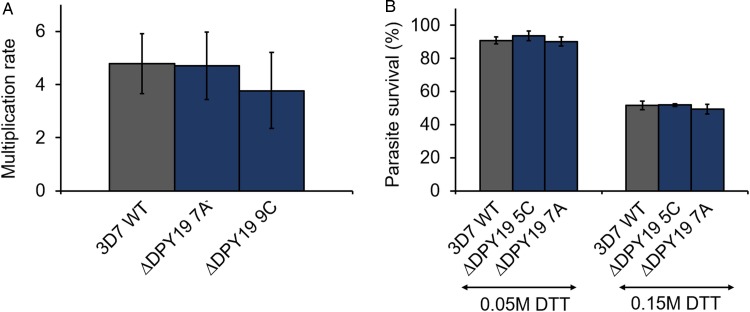
*Pf*DPY19 disruption does not alter parasite growth. (A) The growth rate of 3D7 1.2B wild-type (3D7 WT) parasites and *Pf*DPY19 null mutants (ΔDPY19 7A and 9C clones) was monitored over a complete intraerythrocytic cycle. Values are the mean of three biological replicates and error bars represent the standard variation. (B) Growth inhibition of 3D7 1.2B wild-type (3D7 WT) parasites and *Pf*DPY19 null mutants (ΔDPY19 5C and 7A clones) at two DTT concentrations (0.05 and 0.15 M). Values are the mean of three technical replicates and error bars represent the standard deviation.

*Plasmodium falciparum* expresses three TSR domain-containing proteins during the blood stage development of the parasite, all of which contain at least one TSR domain that conserves the apicomplexan *C*-mannosylation consensus sequence W-X-X-W-X-X-C (see Table 1). Specifically, antibodies raised against TRAMP block the erythrocyte invasion of *P. falciparum* merozoites (Siddiqui *et al*., 2013). To assess if *Pf*DPY19 gene disruption could have an impact on the secretion of TRAMP (or other acceptors) and, consequently, on parasite growth, we compared the multiplication rate of *Pf*DPY19 null mutants with that of the parental wild-type line (Fig. 2A). However, we did not observe any difference across three biological replicates, suggesting that *Pf*DPY19 disruption does not affect the parasite's asexual blood stage *in vitro* growth rate.

During the intraerythrocytic development, the parasite is exposed to oxidative stress due to the host immune responses and the products generated during haemoglobin degradation. Thus, the parasite is especially vulnerable to redox imbalances that affect protein folding in the ER (Chaubey *et al*., 2014). Given the alleged role of *C-*mannosylation in the stabilization of the tryptophan ladder of TSR domains (Buettner *et al*., 2013; Shcherbakova *et al*., 2017), we compared the survival rate of *Pf*DPY19 null mutants with that of the parental wild-type line when subjected to different concentrations of DTT (Fig. 2B) to assess the impact of the absence of a *Pf*DPY19 *C*-mannosyltransferase activity when coping with such conditions. However, no significant differences were observed at any of the DTT concentrations tested.

## Discussion

The recent functional characterization of *P. falciparum* PoFUT2 suggests a critical role of *O*-fucosylation during the infection of the mosquito host (Lopaticki *et al*., 2017), although these results could not be replicated in an independent study (Sanz *et al*., 2019). According to the work conducted by Lopaticki *et al*., PoFUT2 disruption results in a deficient secretion of TRAP, which probably affects the parasite ability to invade hepatocytes. Moreover, it is likely that other acceptors are also affected by the absence of *O*-fucosyltransferase activity, as evidenced by the deficient invasion of the mosquito midgut epithelium. Although TRAP has also been demonstrated to be *C*-mannosylated in salivary gland sporozoites (Swearingen *et al*., 2016), the relevance of this modification remains unknown. The essential role of *C-*mannosylation for protein secretion observed in other organisms (Buettner *et al*., 2013; Shcherbakova *et al*., 2017) prompted us to investigate the contribution of *Pf*DPY19 to parasite survival and infectivity.

Contrary to *O*-fucosylation (Lopaticki *et al*., 2017; Sanz *et al*., 2019), *C*-mannosylation is also expected to occur during the blood stages of the parasite, as supported by the conservation of the apicomplexan consensus sequence (Hoppe *et al*., 2018) in the TSR domain-containing proteins PTRAMP, MTRAP and SPATR (Table 1). However, the successful generation of a parasite line with a severely truncated *Pf*DPY19 gene strongly suggests that this modification is not essential for the asexual development of the parasite. Furthermore, parasite growth was not affected in Δ*Pf*DPY19 mutants, suggesting that the gene is not relevant during the asexual multiplication of the parasite, at least under the tested conditions. This finding is in partial agreement with a previous high-throughput screening study that described the gene coding for *Pf*DPY19 as dispensable, but conferring significant fitness to *P. falciparum* in the asexual blood stages (Zhang *et al*., 2018). The discrepancy might arise from slight deviations associated with the comparison of sequencing reads as a proxy for competitive growth fitness in this particular gene or genomic region. The contribution of *Pf*DPY19 to the secretion of the putative acceptors expressed in the blood stages has not been directly assessed. Nevertheless, if there is an effect on protein secretion secondary to *Pf*DPY19 gene disruption, it does not seem to affect neither parasite growth nor the parasite's ability to cope with DTT-induced oxidative stress. Contrary to this, a recent work reports that *Toxoplasma gondii* DPY19 may be important for the growth fitness of *T. gondii* tachyzoites, a related apicomplexan (Gas-Pascual *et al*., 2019).

The *O-*fucosylation dependence for protein secretion seems to be variable among the different acceptors that are expressed in the mosquito stages of *P. falciparum* (Lopaticki *et al*., 2017). These findings are in agreement with similar observations made with human TSR domain-containing proteins after the loss of the enzyme responsible for *O*-fucose elongation with glucose (Vasudevan *et al*., 2015). Similarly, *C*-mannosylation seems to be required for the secretion of only a subset of its acceptors in *C. elegans* (Buettner *et al*., 2013; Shcherbakova *et al*., 2017). Thus, this modification may be more relevant in other stages of the parasite, where other putative effectors have also been described to play essential roles for parasite viability and infectivity. For instance, given MTRAP essentiality for gametocyte egress (Bargieri *et al*., 2016), a reduced secretion of this protein may result in a deficient transmission to the mosquito. Likewise, a reduced secretion of CTRP or TRAP as it has been reported after *Pf*PoFUT2 gene disruption (Lopaticki *et al*., 2017) may cause a deficient mosquito midgut colonization and hepatocyte invasion, respectively. Furthermore, specific *in vivo* environmental conditions, such as temperature variations or other ER stress inducers, might affect the secretion of different acceptors along the parasite life cycle, including the asexual blood stages. Hence, further studies are required to define the function of *Pf*DPY19 *C*-mannosyltransferase in *Plasmodium* parasites.
